# Long-term forgetting is independent of age in healthy children and adolescents

**DOI:** 10.3389/fpsyg.2024.1338826

**Published:** 2024-06-03

**Authors:** Felizia Pellegrini, Nina Uebelhardt, Sandra Bigi, Martina Studer, Luana Nocco, Kevin Wingeier, Karen Lidzba

**Affiliations:** ^1^Division of Neuropediatrics, Development and Rehabilitation, Department of Pediatrics, Inselspital, Bern University Hospital, University of Bern, Bern, Switzerland; ^2^Institute for Social and Preventive Medicine, University of Bern, Bern, Switzerland; ^3^Division of Pediatric Neurology, Department of Pediatrics, Children’s Hospital Lucerne, Lucerne, Switzerland; ^4^Department of Pediatric Neurology and Developmental Medicine, University of Basel Children’s Hospital, Basel, Switzerland; ^5^Department of Psychosomatics and Psychiatry, University Children's Hospital Zurich, Zurich, Switzerland

**Keywords:** long-term memory, forgetting, intelligence, development, accelerated long-term forgetting

## Abstract

**Introduction:**

In clinical neuropsychology, the phenomenon of accelerated long-term forgetting (ALF) has advanced to be a marker for subtle but clinically relevant memory problems associated with a range of neurological conditions. The normal developmental trajectory of long-term memory, in this case, memory recall after 1 week, and the influence of cognitive variables such as intelligence have not extensively been described, which is a drawback for the use of accelerated long-term forgetting measures in pediatric neuropsychology.

**Methods:**

In this clinical observation study, we analyzed the normal developmental trajectory of verbal memory recall after 1 week in healthy children and adolescents. We hypothesized that 1-week recall and 1-week forgetting would be age-dependent and correlate with other cognitive functions such as intelligence and working memory. Sixty-three healthy participants between the ages of 8 and 16 years completed a newly developed auditory verbal learning test (WoMBAT) and the WISC-V intelligence test (General Ability Index, GAI). Using these tests, 1 week recall and 1 week forgetting have been studied in relation to GAI, verbal learning performance, and verbal working memory.

**Results:**

Neither 1-week recall nor 1-week forgetting seems to be age-dependent. They are also not significantly predicted by other cognitive functions such as GAI or working memory. Instead, the ability to recall a previously memorized word list after 7 days seems to depend solely on the initial learning capacity.

**Conclusion:**

In the clinical setting, this finding can help interpret difficulties in free recall after 7 days or more since they can probably not be attributed to young age or low intelligence.

## 1 Introduction

The ability to store and successfully retrieve verbal information in long-term memory is crucial for children and adolescents, not only for academic achievement. Morphological and functional maturation of the hippocampus, which is essential for episodic information recall, is protracted into the early (Eckenhoff and Rakic, 1991) to middle (Paz-Alonso et al., 2013) school years, suggesting functional changes until this time. In addition, a range of developmental processes lead to an increase in memory capacity during childhood and adolescence: The rehearsal process is rendered more efficient by an increase in processing speed (Schneider and Bjorklund, 2003), while memory encoding profits from the maturation of attention processes and executive functions (Raj and Bell, 2010). Consequently, during childhood and adolescence, the norms of classical list-learning tests reflect an age-dependent increase in the number of words encoded and recalled. The classic theories on the dynamics of forgetting predict that most loss occurs immediately after encoding, within minutes or hours (Anderson and Schooler, 1991). At longer intervals, the process of forgetting more or less plateaus so that differences in forgetting diminish from weeks to a year (Cepeda et al., 2008). While this dynamic can be taken for granted in healthy adults, it seems to be altered in specific patient groups, leading to a clinical phenomenon termed “accelerated long-term forgetting” (ALF; Blake et al., 2000; Fitzgerald et al., 2013). The term is used whenever information is encoded correctly in the first place and remembered correctly after a short time interval (e.g., 30 min) but is then forgotten at an unusually rapid speed over the course of days to weeks (Elliott et al., 2014; Cassel and Kopelman, 2019). ALF has mostly been linked to epilepsy, but it might be a general, sensitive measure to detect subtle but clinically relevant memory deficits irrespective of the underlying neurologic condition, as already demonstrated in patients with other conditions such as preclinical Alzheimer’s disease, traumatic brain injury, or transient ischemic attacks (Mameniskiene et al., 2020). The phenomenon may be apt to explain patients’ everyday memory complaints despite within age-norm performance in the regular clinical memory tests covering only short periods of delayed recall (Fitzgerald et al., 2013; Mameniskiene et al., 2020). So far, research on ALF focused mainly on adult patients (Elliott et al., 2014). Literature describing ALF in the pediatric age group is scarce (Guimarães et al., 2007; Kernan et al., 2012; Gascoigne et al., 2014, 2019; Stahli et al., 2022; Studer et al., 2023), although ALF could have dramatic effects on children’s academic performance. Currently, however, standardized memory tests cannot capture ALF in the pediatric population since there is little knowledge about the normal developmental dynamics and the neuropsychological factors affecting memory recall and loss after more than 30 min. Since the outcome of classical memory studies (e.g., Anderson and Schooler, 1991) does not suggest large population differences in the forgetting rate, it might be assumed that this holds true also for younger vs. older children. However, this general assumption is challenged by the abovementioned studies on ALF in specific patient groups, and thus, differences between age groups should not be ruled out from the start. Although some studies on ALF in children did compare patient data to that of typically developing controls, specific age effects have rarely been reported. In a story learning task, children between 4 and 11 years got more efficient during the trials, leading to higher rates of free recall after 1 week in older children. In this group, story recall was not influenced by intelligence but by verbal fluency and verbal working memory (Van Iterson, 2019). For word list learning tasks, however, little is reported on the proportion of words being forgotten after a period of time, and age-appropriate standard scores for this parameter are often lacking (Strauss et al., 2006). Healthy children and adolescents between 6 and 16 years have been reported to recall approximately 74% after having reached the criterion of 100% during the learning trials a week before (Gascoigne et al., 2014, 2019). Interestingly, a significant age effect for 1-week recall was detected in the group of children with epilepsy but not in the control group of 58 typically developing children (Gascoigne et al., 2014). In many other studies on free memory recall after longer time intervals, the performance of healthy children is, unfortunately, not explicitly stated (Gascoigne et al., 2012; Lah et al., 2017; Grayson-Collins et al., 2019; Joplin et al., 2020), and age effects within the healthy group have, in general, not been reported. For the clinical assessment of longer recall and forgetting intervals in the pediatric population, however, sound age-appropriate normative data are even more important than they might be in the adult population (American Academy of Clinical Neuropsychology, 2007). Therefore, a study dedicated to memory recall and forgetting over a period of days or weeks in typically developing children and adolescents is dearly needed. While the aim of our main project was to assess long-term memory and forgetting, especially ALF, in children with neuropediatric diseases, this study presents information on what to expect in typically developing children. The scope of this study was to analyze the developmental trajectory and potential neuropsychological predictors of recall and forgetting after 1 week in healthy children and adolescents. To provide data that can ultimately inform clinical practice, we used a test based on one of the most commonly used paradigms for episodic memory recall and forgetting, namely the Rey Auditory Verbal Learning Test (Schmidt, 1996). Many experimental tests have adopted the same principle but added a delayed recall after 1 week (Tremont et al., 2000). The operationalization of forgetting varies both in clinical assessments and in experimental studies. While some groups focusing on the theoretical processes of learning and memory in healthy adults have established sophisticated methods to achieve individual forgetting rates (e.g., Sense et al., 2016), these methods are, for several reasons, not feasible under clinical conditions with pediatric patients. Under clinical conditions, we are facing a high variance in the learning rate, in addition to time constraints due to structural (hospital economics) and patient-related (attention span) factors. Many experiments study memory and forgetting and use recognition to determine the efficacy of memory processes. While recognition is less dependent on executive functions in terms of individual strategies than free recall, the classical decision tasks (old vs. new item) have been strongly criticized (Brady et al., 2023). Furthermore, patients with epilepsy do not commonly differ from healthy controls in their recognition performance (Davidson et al., 2007; Schraegle et al., 2016; Studer et al., 2023). We aimed to develop a test suitable for the clinical context, meeting the time constraints due to economic factors and the individually variable capacities of the patients. We adopted the principle of word list learning, using a new experimental auditory verbal learning and memory test (“Wortlisten Merken, Behalten und Abrufen Test”; WoMBAT; Nocco et al., in preparation), containing words suitable for German-speaking children and adolescents. We opted for a fixed set of learning trials instead of the experimentally more common principle of learning to criterion. Forgetting was operationalized by the proportion of words forgotten after 1 week as compared to words retained after 30 min. With this measure, we tried to meet the special conditions of clinical application in patient groups with variable cognitive potentials, accepting the statistical drawbacks resulting from the non-linearity of the underlying forgetting process.

We hypothesized that (1) older children and adolescents have better 1-week recall performance than younger children; and (2) verbal working memory and verbal learning rate, but not intelligence, have an influence on both 1-week recall and forgetting.

## 2 Materials and methods

### 2.1 Participants

Participants were recruited as part of two larger, ongoing studies between November 2019 and March 2021 at the University’s Children Hospital Bern, both approved by the cantonal ethics committee of Bern, Switzerland (2019–01454 and 2020–00596). The focus of these larger studies lay on the assessment of long-term memory in two clinical neuropediatric populations, namely children with epilepsies and children after traumatic brain injury. In this study, we report data from the control groups. Eligible participants for these control groups (a) were healthy and without any neurological or psychiatric condition such as TBI, epilepsy, attention deficit disorder, or autism spectrum disorder; (b) were aged 8–16 years; (c) were proficient in German; (d) did not have any signs of language problems; (e) did not have any severe visual or hearing impairments; (f) were enrolled in a regular school program; and (g) had an IQ within two standard deviations of the mean (i.e., IQ between 70 and 130).

### 2.2 Study procedure

The assessments were conducted by trained research assistants (supervised by certified neuropsychologists) either at the University Children’s Hospital Bern (Inselspital) or at participants’ homes. In both settings, care was given to the environment, which was quiet and free of distraction during the testing procedure. Participants were compensated with a media voucher, according to the time dedicated. All participants were tested with the Wechsler Intelligence Scale for Children (WISC-V), a newly developed auditory verbal learning and memory test (“Wortlisten Merken, Behalten und Abrufen Test”; WoMBAT; Nocco et al. in prep.; Studer et al., 2023), a computerized version of a Symbol-Digit-Modalities Test (c-SDMT) (Bigi et al., 2017) and a self-created questionnaire about cognitive and social performance of daily living, rated by parents and children. For the analyses of interest, only data from WISC-V and WoMBAT are considered. We operationalized the intelligence construct by the General Ability Index (GAI; consisting of the subtests block design, similarities, matrix reasoning, vocabulary, and figure weights) instead of the Full Scale IQ (FS IQ) since the latter depends on working memory performance. For the WoMBAT, participants had to memorize a word list of 17 words over four learning runs. The word list has been developed according to the following criteria: (1) Only nouns in the singular form were used; (2) All words were part of the basic German lexicon (Grund, 1996) and evaluated for child-friendliness; (3) All words were of high frequency, according to two established lexica (Universität Leipzig, 2011; Leibniz Institut Für Deutsche Sprache, 2012); (4) Within the list, semantic independence between the words was assured; (5) Phonemic features, such as initial letters and word-endings, were equally distributed within the list; and (6) Word sequence was thoroughly checked to avoid obvious semantic bridges between neighboring words. After each run, participants were asked to repeat the words aloud (T1–T4). The sum of all words recalled after each learning run was noted as the verbal learning rate. After the learning runs, a second list of 17 words (interference; I) was read to and repeated by the participant. The interference list was constructed according to the same principles as the main list. Both lists are comparable with respect to (1) the number of syllables and letters, (2) the use of semantic categories, (3) phonemic features, and (4) the gender of the nouns (masculine, feminine, and neutral). After the interference list, participants were asked for a free recall of as many words as possible from the first list (T5). Furthermore, two announced delayed free recalls were asked from the participants both after 30 min (T6) and after 7 days (T7). For the 1-week recall, all participants were called by phone.

## 3 Results

### 3.1 Data analysis plan

IBM SPSS Statistics (Version 25) has been used for statistical analyses. A *p*-value of <0.05 was considered statistically significant, and Bonferroni correction was applied in case of multiple testing. The potential effect of sex on the WoMBAT parameters was first examined by multiple T-tests (see Supplementary Table S1). Since for some of the parameters of interest, namely learning ability and recall loss, the difference was significant or approached significance, we introduced sex as a covariate in all analyses.

To test hypothesis 1, we conducted partial correlations between age (years) and verbal learning rate (∑(T1,T2,T3,T4)), between age (years) and free recall after 30 min (T6), between age (years) and free recall after 7 days (T7), and between age (years) and recall loss between free recall after 30 min and free recall after 7 days (100%*(T6-T7)/T6), respectively. All correlations were corrected for sex (male/female) if parametric testing was possible. Power analysis indicated that a sample size of *N* = 68 would be needed to detect a medium effect of 0.3 with a power of 0.80 in these correlations.

Potential predictors for long-term memory and long-term forgetting were examined with two hierarchical linear regression analyses, introducing age (years) and intelligence (WISC-V General Ability Index) in a first block of predictors, WoMBAT verbal learning rate (∑(T1, T2, T3, T4)) in a second block of predictors, and working memory (WISC-V Digit Span) as predictor of interest. R2 change was used as an indicator for individual variance explained by every new predictor. As a dependent variable, we first used free recall after 7 days (T7), and, in a second analysis, recall loss after 7 days (100%*(T6-T7)/T6). Power analysis indicated that a sample size of *N* = 49 to *N* = 70 would be needed to detect a small-to-medium effect of 0.2–0.3 with a power of 0.80 in these regression analyses.

### 3.2 Descriptive data

Complete WoMBAT data were available for *n* = 7 participants. After inspection of data quality, one outlier was removed from the dataset due to implausible performance (a 9-year-old male with virtually no successful learning trial but perfect free recall after 7 days). WISC-V data were missing for *n* = 4 individuals. *N* = 6 participants were excluded due to GAI scores >130. Descriptive statistics of the sample are shown in Table 1. Since the variables age, GAI, and recall loss are not normally distributed (Shapiro–Wilk test), we used Spearman–Rank correlations for the correlational analyses. This procedure does not allow for the introduction of covariates. Differential effects of sex on the WoMBAT parameters are shown in the Supplementary Table S1. In short, only verbal learning rates differed significantly between boys and girls (males: mean = 42.54, *sd* = 7.43; females: mean 46.95, *sd* = 7.42; *p* = 0.02, *t*-test).

**Table 1:** Sample characterization.

Sex; male/female *n* = 67	28/39
Age in years; *n* = 67; mean (range)	11.82 (8–16)
WISC-V General Ability Index; *n* = 63; mean (sd; range)	112.37 (12.71; 82–129)
WISC-V Digit Span Scaled Score; *n* = 63; mean (sd; range)	11.83 (2.62; 4–19)
WoMBAT verbal learning rate (∑(T1,T2,T3,T4)); *n* = 67; mean (sd; range)	45.10 (7.69; 26–65)
WoMBAT free recall 30 min (T6); *n* = 67; mean (sd; range)	11.81 (3.04; 3–17)
WoMBAT free recall 7 days (T7); *n* = 67; mean (sd; range)	9.34 (3.32; 2–16)
WoMBAT recall loss 7 days (100%*(T6-T7)/T6); *n* = 67; mean (sd; range)	29.27% (24.03; −60% −71.43%)

### 3.3 Age-effects

**Figure 1:**
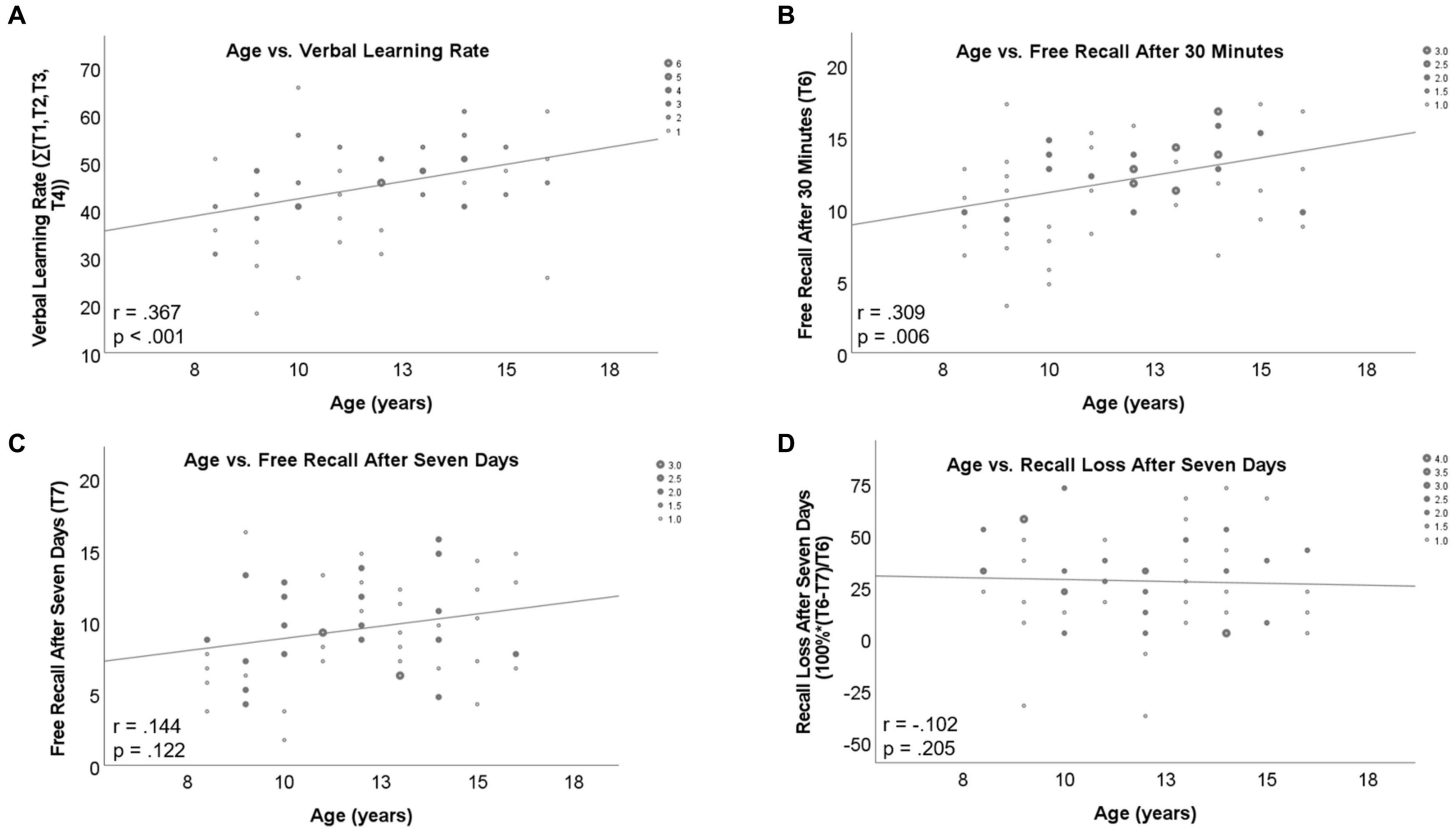
Correlations between age and WoMBAT parameters.

As expected, age correlated significantly (Spearman Rank; one-tailed; Bonferroni-corrected *p* = 0.01) with verbal learning rate (*r* = 0.367, *p* < 0.001; Figure 1A) and free recall after 30 min (*r* = 0.309, *p* = 0.006; Figure 1B). Correlation of age with free recall after 7 days missed significance (*r* = 0.144, *p* = 0.122; Figure 1C). Recall loss was not significantly correlated with age (*r* = −0.102, *p* = 0.205; Figure 1D).

### 3.4 Effects of intelligence and working memory

The first hierarchical regression analysis (Table 2) revealed that age and GAI do not significantly predict free recall after 7 days (corrected *R^2^* = 0.02; *p* = 0.208). The amount of explained variance significantly increases with the introduction of verbal learning rate (by 14%; *p_change_* = 0.003). Working memory does not significantly add to the amount of variance explained (*p_change_* = 0.142). The second and the third model were similarly meaningful (Model 2: *F_3,62_* = 4.48; *p* = 0.007; Model 3: *F_4,62_* = 3.98; *p* = 0.006).

**Table 2:** Hierarchical regression analyses on the predictors of free recall after 7 days (*n* = 63).

	Predictors added to the model	Standardized *β*	*p* value	Corrected *R^2^*	*R^2^* change	*p* change
1	Age WISC-V General Ability Index	0.08 −0.06	0.49 0.64	0.06	0.02	0.208
2	WoMBAT verbal learning rate	0.45	<0.001	0.23	0.14	0.003
3	WISC-V digit span	0.18	0.123	0.24	0.03	0.142

The second hierarchical regression analysis (Table 3) revealed that age and GAI do not significantly predict recall loss (*p* = 0.602). The amount of explained variance does not significantly increase with the introduction of verbal learning rate (*p_change_* = 0.847). Working memory does not significantly add to the amount of variance explained (*p_change_* = 0.101). None of the models reached statistical significance.

**Table 3:** Hierarchical regression analyses on the predictors of recall loss after 7 days (*n* = 63).

	Predictors added to the model	Standardized *β*	*p* value	Corrected *R^2^*	*R^2^* change	*p* change
1	Age WISC-V General Ability Index	−0.08 0.19	0.57 0.17	−0.01	0.02	0.602
2	WoMBAT learning ability	0.08	0.54	−0.02	0.00	0.847
3	WISC-V digit span	−0.22	0.11	0.01	0.04	0.101

## 4 Discussion

The main objective of the present study is to explore the developmental trajectory of long-term memory in healthy individuals between the ages of 8 and 16 years. More precisely, 1-week recall and 1-week forgetting have been studied in relation to intelligence, verbal learning rate, and verbal working memory. Counter to our hypothesis and clinically relevant is the result that free recall and, even more so forgetting, of learned facts after 1 week do not seem to be significantly age-dependent in healthy children and adolescents within the studied age range. Since the correlation between age and free recall after 1 week had a low-to-medium effect size but closely missed significance, we tried a post-hoc analysis to corroborate the presumed trend. However, our non-parametric median test (Fisher exact) between the two youngest and the two oldest age groups clearly suggested keeping the null hypothesis. Free recall and forgetting after 1 week are also not significantly predicted by other cognitive functions such as intelligence or working memory. Instead, the ability to memorize a word list for a short period of time (i.e., verbal learning rate) largely predicts the number of words recalled after 7 days. The more words an individual is able to memorize and recall in the short and medium time period, the more they will remember after a longer interval of time. Forgetting, on the other side, was not predicted by the verbal learning rate in our sample: The proportion of items lost after 1 week seems to be independent of the absolute amount of items acquired. This is in line with classical theories of memory and forgetting, which predict largely uniform and flat individual forgetting curves after an initial period of some hours (Cepeda et al., 2008). In a younger cohort of children between 4 and 10 years, however, significant effects of age and intelligence have been reported for delayed free recall of a repeatedly learned story (Van Iterson, 2019). Thus, from a clinical perspective, it is important to evaluate potential age effects on the forgetting process also during the plateau phase. The ability to remember a story increases not only with increasing memory capacity but is facilitated by the ability to intellectually integrate the story content, which should be easier for individuals with higher verbal intelligence. Encoding semantically incoherent words, on the other hand, is more closely related to mere memory capacity and may rely more on executive functions or meta-cognitive abilities. Concordantly, free long-term recall of word lists was uncorrelated with intelligence in clinical samples of children with traumatic brain injury (Lah et al., 2017) or epilepsy (Grayson-Collins et al., 2019). Although we did not find an impact of working memory on very long-term memory or recall loss in our sample of typically developing children, a range of executive functions have been significantly associated with verbal memory recall after 1 week in clinical samples (Van Iterson, 2019; Joplin et al., 2022; Studer et al., 2023). In a mixed group of children with and without epilepsy, story recall was positively associated with verbal fluency and verbal working memory (Van Iterson, 2019). Word list recall was positively correlated with both verbal and figural fluency but not with verbal working memory in a group of children with epilepsy (Studer et al., 2023).

### 4.1 Limitations

The main limitation of our study is the relatively small sample size, which included 63 participants. Due to lockdown measures during the COVID-19 pandemic, recruitment was hindered, and thus, we could not achieve the originally planned sample size of *n* = 100. This may have led to statistical underpowering in some aspects. The study sample was too small to explore all possible interactions between the various cognitive and demographic factors, such as the children’s socio-economic background or the educational background of their parents. It is also imaginable that significant effects of age or intelligence on 1-week recall or 1-week forgetting might be revealed in a cohort larger than the 63 participants we studied. Furthermore, we acknowledge that the memory paradigm we chose for this study has a range of drawbacks. With a more elaborate paradigm employing more stimuli and a two-alternative forced-choice recognition task in addition to the free recall, we could have made individual ROC analyses, leading to more precise parameters of forgetting. However, aiming at a memory paradigm that can be readily employed in clinical practice, time-on-test is a crucial factor. Thus, we opted for the clinically established variant. Due to lockdown periods during the COVID-19 pandemic, some participants were tested at home, and others were tested at the hospital. Both surroundings bear potential distractors, which we tried to prevent as well as possible. A setting with some distracting stimuli (such as noise from helicopters or from children in the corridor in the hospital, and noise from siblings playing around the house in the home setting) is a clinical reality, and so we are not concerned too much by this variance in test settings. Finally, it is important to note that the average performance in the WISC-V was relatively high (although within the normal range) and that a greater diversity might have led to different results.

## 5 Conclusion

Still, our study results have practical clinical implications: Clinicians working with children and adolescents are used to strong age effects in all kinds of neuropsychological functions. They are also cautious when interpreting specific neuropsychological functions in the light of general intelligence. Thus, knowledge about the absence of the effects of age and intelligence with respect to 1-week forgetting is extremely important since clinicians might be in danger of misinterpreting ALF as a developmentally appropriate phenomenon in younger children or those with intellectual disability. Additionally, on the pragmatic side, the long-term recall loss in clinical memory tests can be standardized age-independently (at least between the age of 8–16 years), leading to a more robust comparison group and/or more economical test development.

## Data availability statement

The raw data supporting the conclusions of this article will be made available by the authors, without undue reservation.

## Ethics statement

The studies involving humans were approved by Kantonale Ethikkommission Bern. The studies were conducted in accordance with the local legislation and institutional requirements. Written informed consent for participation in this study was provided by the participants’ legal guardians/next of kin.

## Author contributions

FP: Data curation, Investigation, Writing – original draft, Writing – review & editing. NU: Data curation, Investigation, Writing – original draft, Writing – review & editing. SB: Conceptualization, Methodology, Supervision, Writing – review & editing. MS: Funding acquisition, Supervision, Writing – review & editing. LN: Writing – review & editing. KW: Methodology, Supervision, Writing – review & editing. KL: Conceptualization, Formal analysis, Funding acquisition, Investigation, Methodology, Project administration, Resources, Supervision, Writing – review & editing.

## References

[ref1] American Academy of Clinical Neuropsychology (2007). American Academy of clinical neuropsychology (Aacn) practice guidelines for neuropsychological assessment and consultation. Clin. Neuropsychol. 21, 209–231. doi: 10.1080/13825580601025932, PMID: 17455014

[ref2] Anderson J. R. Schooler L. J. (1991). Reflections of the environment in memory. Psychol. Sci. 2, 396–408. doi: 10.1111/j.1467-9280.1991.tb00174.x

[ref3] Bigi S. Marrie R. A. Till C. Yeh E. A. Akbar N. Feinstein A.. (2017). The computer-based symbol digit modalities test: establishing age-expected performance in healthy controls and evaluation of pediatric Ms patients. Neurol. Sci. 38, 635–642. doi: 10.1007/s10072-017-2813-0, PMID: 28078569

[ref4] Blake R. V. Wroe S. J. Breen E. K. Mccarthy R. A. (2000). Accelerated forgetting in patients with epilepsy: evidence for an impairment in memory consolidation. Brain 123, 472–483. doi: 10.1093/brain/123.3.472, PMID: 10686171

[ref5] Brady T. F. Robinson M. M. Williams J. R. Wixted J. T. (2023). Measuring memory is harder than you think: how to avoid problematic measurement practices in memory research. Psychon. Bull. Rev. 30, 421–449. doi: 10.3758/s13423-022-02179-w, PMID: 36260270 PMC10257388

[ref6] Cassel A. Kopelman M. D. (2019). Have we forgotten about forgetting? A critical review of 'accelerated long-term forgetting' in temporal lobe epilepsy. Cortex 110, 141–149. doi: 10.1016/j.cortex.2017.12.012, PMID: 29331203

[ref7] Cepeda N. J. Vul E. Rohrer D. Wixted J. T. Pashler H. (2008). Spacing effects in learning: a temporal ridgeline of optimal retention. Psychol. Sci. 19, 1095–1102. doi: 10.1111/j.1467-9280.2008.02209.x 19076480

[ref8] Davidson M. Dorris L. O’regan M. Zuberi S. M. (2007). Memory consolidation and accelerated forgetting in children with idiopathic generalized epilepsy. Epilepsy Behav. 11, 394–400. doi: 10.1016/j.yebeh.2007.05.004, PMID: 17715001

[ref9] Eckenhoff M. F. Rakic P. (1991). A quantitative analysis of synaptogenesis in the molecular layer of the dentate gyrus in the rhesus monkey. Brain Res. Dev. Brain Res. 64, 129–135. doi: 10.1016/0165-3806(91)90216-6, PMID: 1786637

[ref10] Elliott G. Isaac C. L. Muhlert N. (2014). Measuring forgetting: a critical review of accelerated long-term forgetting studies. Cortex 54, 16–32. doi: 10.1016/j.cortex.2014.02.001, PMID: 24631847 PMC4007031

[ref11] Fitzgerald Z. Mohamed A. Ricci M. Thayer Z. Miller L. (2013). Accelerated long-term forgetting: a newly identified memory impairment in epilepsy. J. Clin. Neurosci. 20, 1486–1491. doi: 10.1016/j.jocn.2013.04.037, PMID: 24076316

[ref12] Gascoigne M. B. Barton B. Webster R. Gill D. Antony J. Lah S. S. (2012). Accelerated long-term forgetting in children with idiopathic generalized epilepsy. Epilepsia 53, 2135–2140. doi: 10.1111/j.1528-1167.2012.03719.x, PMID: 23061735

[ref13] Gascoigne M. B. Smith M. L. Barton B. Webster R. Gill D. Lah S. (2014). Accelerated long-term forgetting in children with temporal lobe epilepsy. Neuropsychologia 59, 93–102. doi: 10.1016/j.neuropsychologia.2014.04.012, PMID: 24784007

[ref14] Gascoigne M. B. Smith M. L. Barton B. Webster R. Gill D. Lah S. (2019). Accelerated long-term forgetting and behavioural difficulties in children with epilepsy. Cortex 110, 92–100. doi: 10.1016/j.cortex.2018.03.021, PMID: 29685768

[ref15] Grayson-Collins J. Gascoigne M. B. Barton B. Webster R. Gill D. Lah S. (2019). Longitudinal study of accelerated long-term forgetting in children with genetic generalized epilepsy: evidence of ongoing deficits. Cortex 110, 5–15. doi: 10.1016/j.cortex.2017.08.028, PMID: 28988644

[ref16] Grund M. (1996). Grundwortschatz Training Gut1. Available at: www.gut1.de (Accessed January 12, 2019).

[ref17] Guimarães C. A. Li L. M. Rzezak P. Fuentes D. Franzon R. C. Augusta Montenegro M.. (2007). Temporal lobe epilepsy in childhood: comprehensive neuropsychological assessment. J. Child Neurol. 22, 836–840. doi: 10.1177/0883073807304701, PMID: 17715275

[ref18] Joplin S. Gascoigne M. Barton B. Webster R. Gill D. Lawson J. A.. (2022). Accelerated long-term forgetting in children with temporal lobe epilepsy: a timescale investigation of material specificity and executive skills. Epilepsy Behav. 129:108623. doi: 10.1016/j.yebeh.2022.108623, PMID: 35259627

[ref19] Joplin S. Webster R. Gill D. Barton B. Lawson J. A. Mandalis A.. (2020). Accelerated long-term forgetting in children with genetic generalized epilepsy: the temporal trajectory and contribution of executive skills. Epilepsy Behav. 113:107471. doi: 10.1016/j.yebeh.2020.107471, PMID: 33142199

[ref20] Kernan C. L. Asarnow R. Siddarth P. Gurbani S. Lanphier E. K. Sankar R.. (2012). Neurocognitive profiles in children with epilepsy. Epilepsia 53, 2156–2163. doi: 10.1111/j.1528-1167.2012.03706.x 23126490

[ref9001] Lah S. Black C. Gascoigne M. B. Gott C. Epps A. Parry L. (2017). Accelerated long-term forgetting is not epilepsy specific: evidence from childhood traumatic brain injury. J. Neurotrauma. 34, 2536–2544. 28482744 10.1089/neu.2016.4872

[ref21] Leibniz Institut Für Deutsche Sprache (2012). Grundformliste. Available at: http://www1.idsmannheim.de/kl/projekte/methoden/derewo.html

[ref22] Mameniskiene R. Puteikis K. Jasionis A. Jatuzis D. (2020). A review of accelerated long-term forgetting in epilepsy. Brain Sci. 10:945. doi: 10.3390/brainsci10120945, PMID: 33297371 PMC7762289

[ref23] Paz-Alonso P. M. Gallego P. Ghetti S. (2013). Age differences in hippocampus-cortex connectivity during true and false memory retrieval. J. Int. Neuropsychol. Soc. 19, 1031–1041. doi: 10.1017/S1355617713001069, PMID: 24060006

[ref24] Raj V. Bell M. A. (2010). Cognitive processes supporting episodic memory formation in childhood: the role of source memory, binding, and executive functioning. Dev. Rev. 30, 384–402. doi: 10.1016/j.dr.2011.02.001

[ref25] Schmidt M. (1996). Rey auditory verbal learning test: Ravlt: A handbook. Los Angeles: Western Psychological Services.

[ref26] Schneider W. Bjorklund D. (2003). “Memory and knowledge development” in Handbook of developmental psychology. eds. Valsiner J. Connolly K. (London: Sage), 370–403.

[ref27] Schraegle W. A. Nussbaum N. L. Stefanatos A. K. (2016). List-learning and verbal memory profiles in childhood epilepsy syndromes. Epilepsy Behav. 62, 159–165. doi: 10.1016/j.yebeh.2016.07.021, PMID: 27484747

[ref28] Sense F. Behrens F. Meijer R. R. Van Rijn H. (2016). An individual's rate of forgetting is stable over time but differs across materials. Top. Cogn. Sci. 8, 305–321. doi: 10.1111/tops.12183, PMID: 26748838

[ref29] Stahli N. E. Bigi S. Grunt S. Lidzba K. Studer M. (2022). Systematic review of accelerated long-term forgetting in children and adolescents with neuropediatric diseases. Neurol Clin Pract 12, e210–e220. doi: 10.1212/Cpj.0000000000200081, PMID: 36540146 PMC9757117

[ref30] Strauss E. Sherman E. M. S. Spreen O. (2006). A compendium of neuropsychological tests: Administration, norms, and commentary. Oxford: Oxford University Press.

[ref31] Studer M. Schmitt S. Wingeier K. Lidzba K. Bigi S. (2023). Delayed episodic memory recall after one week is associated with executive functions and divided attention in pediatric epilepsy patients. Brain and Development 45, 372–382. doi: 10.1016/j.braindev.2023.03.009, PMID: 37037678

[ref32] Tremont G. Halpert S. Javorsky D. J. Stern R. A. (2000). Differential impact of executive dysfunction on verbal list learning and story recall. Clin. Neuropsychol. 14, 295–302. doi: 10.1076/1385-4046(200008)14:3;1-P;Ft295, PMID: 11262704

[ref33] Universität Leipzig (2011). Deutscher Wortschatz. Available at: http://corpora.uni-leipzig.de/de/res?corpusId=deu_newscrawl_2011 (Accessed January 12, 2019).

[ref34] Van Iterson L. (2019). Story learning test: decelerated learning and accelerated forgetting in children with epilepsy. J. Pediatric Neuropsychol. 5, 133–151. doi: 10.1007/s40817-019-00072-4

