# Supplementary material for: Long-term forgetting is independent of age in healthy children and adolescents
Source: Front Psychol. 2024 Jun 3;15:1338826. doi: 10.3389/fpsyg.2024.1338826 (PMC11182042; doi:10.3389/fpsyg.2024.1338826)
Supplement: Supplementary file 1 [file Table_1.DOCX]

Supplementary Table S1. Effect of sex on WoMBAT parameters

| WoMBAT parameter | male (*n* = 28)  mean (*sd*) | female (*n* = 39)  mean (*sd*) | Significance  (*t*-test, two-tailed) | Effect size  (*Cohen*) |
| --- | --- | --- | --- | --- |
| verbal learning rate (∑(T1,T2,T3,T4)) | 42.54 (7.43) | 46.95 (7.42) | *p* = .02 | *d =* -.55 |
| free recall 30 min (T6) | 12.00 (2.67) | 11.87 (2.91) | *p* = .86 | *d* = -.01 |
| free recall seven days (T7) | 9.21 (3.06) | 9.44 (3.54) | *p* = .79 | *d* = -.08 |
| recall loss seven days (100%*(T6-T7)/T6) | 26.50% (25.68) | 31.25% (22.90) | *p* = .43 | *d* = -.28 |
